# Investigations on anopheline mosquitoes close to the nest sites of chimpanzees subject to malaria infection in Ugandan Highlands

**DOI:** 10.1186/1475-2875-11-116

**Published:** 2012-04-17

**Authors:** Sabrina Krief, Florence Levrero, Jean-Michel Krief, Supinya Thanapongpichat, Mallika Imwong, Georges Snounou, John M Kasenene, Marie Cibot, Jean-Charles Gantier

**Affiliations:** 1 UMR 7206- Eco-anthropologie et ethnobiologie, Musum National dHistoire Naturelle, Paris, France; 2 Projet pour la Conservation des Grands Singes, Paris, France; 3 Universit de Saint-Etienne, Equipe de Neuro-Ethologie Sensorielle/CNPS, CNRS UMR 8195. Centre National de la Recherche Scientifique, Centre de Neurosciences Paris-Sud, UMR 8195, Saint Etienne, France; 4 Faculty of Tropical Medicine, Mahidol University, Bangkok, Thailand; 5 Institut National de la Sant et de la Recherche Mdicale, Unit Mixte de Recherche, S 945 Paris, France; 6 Universit Pierre & Marie Curie, Facult de Mdecine Piti-Salptrire, Paris, France; 7 Makerere University Biological Field Station, Fort Portal, Uganda; 8 Facult de Pharmacie de Chtenay-Malabry, Paris, France

**Keywords:** Malaria, Chimpanzee, *Anopheles*, *Plasmodium*, Kibale National Park, Nesting behaviour

## Abstract

**Background:**

Malaria parasites (*Plasmodium* sp.), including new species, have recently been discovered as low grade mixed infections in three wild chimpanzees (*Pan troglodytes schweinfurthii*) sampled randomly in Kibale National Park, Uganda. This suggested a high prevalence of malaria infection in this community. The clinical course of malaria in chimpanzees and the species of the vectors that transmit their parasites are not known. The fact that these apes display a specific behaviour in which they consume plant parts of low nutritional value but that contain compounds with anti-malarial properties suggests that the apes health might be affected by the parasite. The avoidance of the night-biting anopheline mosquitoes is another potential behavioural adaptation that would lead to a decrease in the number of infectious bites and consequently malaria.

**Methods:**

Mosquitoes were collected over two years using suction-light traps and yeast-generated CO_2_ traps at the nesting and the feeding sites of two chimpanzee communities in Kibale National Park. The species of the female *Anopheles* caught were then determined and the presence of *Plasmodium* was sought in these insects by PCR amplification.

**Results:**

The mosquito catches yielded a total of 309 female *Anopheles* specimens, the only known vectors of malaria parasites of mammalians. These specimens belonged to 10 species, of which *Anopheles implexus*, *Anopheles vinckei* and *Anopheles demeilloni* dominated. Sensitive DNA amplification techniques failed to detect any *Plasmodium*-positive *Anopheles* specimens. Humidity and trap height influenced the *Anopheles* capture success, and there was a negative correlation between nest numbers and mosquito abundance. The anopheline mosquitoes were also less diverse and numerous in sites where chimpanzees were nesting as compared to those where they were feeding.

**Conclusions:**

These observations suggest that the sites where chimpanzees build their nests every night might be selected, at least in part, in order to minimize contact with anopheline mosquitoes, which might lead to a reduced risk in acquiring malaria infections.

## Background

The closest relatives to human beings, chimpanzees, gorillas and orang-utans, are under threat of extinction because of dwindling habitats. The survival of the remaining populations is highly sensitive to the spread of diseases [1]. Great apes are genetically very close to humans and this means that they are susceptible to infection by a variety of pathogens of humans, including bacteria, virus and parasites [2-13]. In this context, malaria is of particular interest. Malaria, a disease that results from infection by a protozoan parasite of the genus *Plasmodium*, is a major global public health problem that undermines development in the poorest countries. For example, in 2008 more than 247 millions cases and one million deaths attributable to malaria were recorded [14]. Three *Plasmodium* species, morphologically very similar to the parasites that infect humans (*Plasmodium vivax* or *Plasmodium ovalePlasmodium malariae* and *Plasmodium falciparum*), have been described early in the 20^th^ century in wild African great apes (chimpanzees, bonobos and gorillas): *Plasmodium schwetziPlasmodium rodhaini*, and *Plasmodium reichenowi*, respectively [15,16]. Two recent molecular surveys of DNA purified from blood samples collected from two chimpanzees kept as pets in Gabon [17], or from three of eight chimpanzees recovered from poachers in the Democratic Republic of Congo (DRC) and in all three wild chimpanzees sampled from Kibale National Park, Uganda [12], uncovered the presence of three more *Plasmodium* species closely related to *P. falciparum*. The fact that all the three chimpanzees sampled randomly among a group of 44 individuals of the Kanyawara community were positive and carried mixed infections suggested that prevalence of the infection might be high in nature. Further surveys conducted on material derived from a large collection of faeces from wild great apes, confirmed the diversity of the parasites in chimpanzees, revealed three additional species related to *P. falciparum* in gorillas, and reinforced the notion that the parasite prevalence could be very high in some communities [18-21]. It is interesting to note that great apes are also susceptible to infection by the parasites of humans. Thus, *P. falciparum* and *P. malariae* were detected in bonobos, cared for in a sanctuary in the Democratic Republic of Congo located within a suburb of Kinshasa, that is endemic for human malaria [12], *P. ovale* was detected in two chimpanzees from Cameroon [22], and finally *P. malariae* was found in two chimpanzees imported to Japan 30years previously [23]. Thus, African great apes harbour a diverse collection of malaria parasites, and are susceptible to infection by those that infect humans.

Kibale National Park (NP) is located in the western highlands of Uganda. The human population in these districts is subjected to malaria such that *Plasmodium* infections are the most common cause of illness (e.g. [24]), as is typically observed in many of the highland districts of Uganda or other sub-Saharan countries [25-35]. It is interesting that malaria has not been recorded as a disease, during extensive long-term monitoring of great apes at Kibale NP and at numerous other research sites. This contrasts with observations of humans in malaria endemic areas. Whether the absence of observed symptoms in wild chimpanzees rests with the substantial difficulties associated with close health monitoring, or to an inherent clinical tolerance in infected apes, or both, remains to be established. The fact that a restricted number of captive chimpanzees experimentally infected in the 1930s and 1940s by a variety of *Plasmodium* species did not suffer severe clinical symptoms [15,16] does not exclude the possibility that chimpanzees experience clinical malaria.

The apparent absence of clinical malaria in natural communities might also be due to non-physiological factors. Two behavioural adaptations might lead to a reduction in malaria. Previous studies have revealed that great apes occasionally consume small amounts of selected parts from several plant species, that have no nutritive value but which contain bio-active compounds with anti-parasitic properties [36-47], including two from *Trichilia rubescens* leaves that are active against *Plasmodium*[48,49]. This provides a tantalizing suggestion that these animals do indeed experience discomfort as a result of malarial infection, which they then seek to alleviate by ingesting specific plants. A second behavioural adaptation would be one of mosquito avoidance. At present, the natural vectors of the parasites that infect the African great apes are not known. All attempts to transmit *P. reichenowi* experimentally by *Anopheles gambiaeAnopheles maculipennis, Anopheles atroparvusAnopheles balabacensisAnopheles freeborni* and *Anopheles stephensi*, failed consistently [16,50-52]. Given the probable high endemicity of malaria in the Kibale chimpanzees, it was felt that investigations of the *Anopheles* mosquitoes in the home ranges of the Kanyawara chimpanzee might help to gather indications whether chimpanzee behaviour alters the nature of the chimpanzee-mosquito contact. It was further hoped that it might also identify which *Anopheles* species naturally transmit *Plasmodium* to chimpanzees. Given that the biting activity in female *Anopheles* generally occurs at night and that proximity of swamp and small variation of temperature may increase malaria risk [53], the present study tested the hypothesis that the choice of chimpanzees for the location and height of their nesting sites may be influenced by the presence of potential vectors of infectious diseases.

## Methods

### Study site

Chimpanzee monitoring and mosquito collections were conducted in both Kanyawara and Kanyanchu areas, in Kibale NP, Western Uganda (0 13-0 41N and 30 19-30 32E). Kibale NP covers an area of 795km^2^ of mild-altitude forest with high biodiversity, most likely because it was a Pleistocene refugium [54]. The area comprises mostly lowland rain forest, montane forest, mixed deciduous forest (57%), colonizing forest (19%), lake and wetlands (2%) with some grassland (15%), and exotic trees plantations (1%) [55]. Some forestry compartments were selectively harvested during the late 1960s [56]. Areas where agricultural activities predominate surround the National Park, thus 58% of the land within 1.4km of the park boundary is used for smallholder agriculture (Mugisha, 1994 cited by [57]).

### Behavioural and clinical observations

Data and mosquitoes were collected in the home ranges of two habituated chimpanzee communities of Kanyawara (44 chimpanzees) and Kanyanchu (120 chimpanzees) in Kibale NP, Uganda. Chimpanzee parties (labile sub-groups of the community in the fission-fusion social system of chimpanzees) were followed daily from nest to nest. Observers were very careful not to disturb chimpanzee behaviour and followed the research proposal reviewed and approved by Uganda Wildlife Authority. Tree species and type of habitat (primary forest or secondary forest) used for nesting, nest height as well as the number and identity of chimpanzees under observation were recorded.

### Ecology and identification of *Anopheles* species in the chimpanzees environment

Mosquitoes were collected throughout the period extending from January 2006 to January 2008 in Kanyawara and from May to July 2009 in Kanyanchu (dry season) using suction light traps (adapted from [58]). At each collection, two traps were used following a protocol (Table1) designed to optimize the trapping and the understanding of mosquito ecology, the mosquito attractant CO_2_[59] was generated by yeast converting sugar in alcohol. The yeast-glucose solution (100g glucose+6g dry yeast in 1.5l of water) was prepared two hours before connection to the trap because the CO_2_ output rate takes 1.5 hour to stabilize [58]. The 2l bottle with the solution was connected with a polypropylene tube to a smaller bottle (0.5l) holding the overflowed solution. The small bottle was then connected to the trap. A 6-volt motor powered by rechargeable batteries drove a fan and diodes. The set-up was covered by a plate of 30cm and helped attract mosquitoes into a net.

**Table 1:** **Protocol followed for *Anopheles *collection in the Kanyawara and Kanyanchu sites, Kibale NP, Uganda**

		**Trap 1**			**Trap 2**	
**Day**	**CO**_**2**_	**Site**	**Trap height**	**CO**_**2**_	**Site**	**Trap height**
**1**	no	Chimpanzees feeding site	2m	no	Chimpanzees nesting site	2m
**2**	no	Chimpanzees nesting site	2m	no	Chimpanzees nesting site	Nest height
**3**	yes	Chimpanzees nesting site	2m	yes	Chimpanzees nesting site	Nest height
**4**	no	village	2m	yes	village	2m

Traps were placed in three different sites to compare features of two types of sites used by chimpanzees (feeding and nesting sites) with control sites during four consecutive days every week: (i) feeding sites used by chimpanzees after 16:30, (ii) nesting sites, (iii) control sites independent of chimpanzee travel (village, hill, swamps). At nesting sites, traps were suspended either at a height of 2m above ground or at the height of one of the nest (up to an height of 17m) and in a close vicinity of the ape (i.e. within a 5m perimeter centred on the tree used by the chimpanzee) (Table1). Traps were switched on late afternoon or evening (16:3019:00) according to feeding and nesting time of the chimpanzees, and switched off and retrieved in the morning (06:3007:30). A total number of 300 traps were placed in Kanyawara and 83 in Kanyanchu.

Altitude (GPS Garmin map 60) of the trapping sites were recorded as well as the temperature and relative humidity, i.e. hygrometry (Oregon Scientific EMR 812), once when the traps were switched on and again when they were switched off (i.e. evening and morning for each trapping site). After trap retrieval, the net was removed then sealed and placed in a big plastic bag (30l) where insects were anesthetized with chloroform. At the research station, mosquitoes were sorted out from other insects and a score ranging from 0 to 3 was determined according to their abundance (1=110; 2=11-20; 3>20). *Anopheles* females were then identified by visual examination, individually counted and then dissected.

Abdomens were stored in 95% ethanol. The head, wings and thorax of each specimen were kept in a dry tube with silica gel to be used for species identification according to identification keys of Gillies and De Meillon [60]. Mosquito collections were carried out regularly throughout the two-year period. Data from traps placed during four consecutive days per week were used to assess the influence of collection conditions (Table1), on the mosquitoes caught.

### Sampling and detection of *Plasmodium* from humans and mosquitoes

Finger-prick blood drops were collected into EDTA tubes, during routine medical diagnosis and treatment by the nurses of Mpanga Tea Factory Dispensary, from 74 informed people (villagers, workers and field assistants) who gave their consent in writing for the study. Formal approval was sought from local and national ethical committees but this was not deemed necessary because the samples were only destined for *Plasmodium* detection. All of persons sampled were at the time working in the Kibale NP, or within less than 1km of the forest border, none were living at more than 15km away from the forest. Of the people sampled, 78% lived less than 2km from the forest and 28% declared they live less than 500m from a swamp. The blood samples were also tested at the time of collection with a Rapid Detection Test (Core DiagnosticTM), and persons found positive were informed of the result and treated appropriately by the medical staff of the Dispensary.

DNA was extracted from finger-prick blood samples and from 100 mosquito carcasses that were kept dry or in ethanol, using the DNeasy Blood & Tissue Kit Qiagen kit (Qiagen, Germany). Six mosquitoes where the species could not be determined were processed individually for DNA extraction, as were 11 *Anopheles demeilloni*, four *Anopheles implexus*, and two *Anopheles vinckei*. For the remaining 77 mosquitoes (10 *A. demeilloni*, 48 *A. implexus*, and 19 *A. vinckei*), three to six (usually five) carcasses were pooled before DNA extraction. In all cases, a final volume of 100l of DNA template was obtained.

Two different sensitive nested PCR protocols were applied to these templates; the first was based on the parasites small subunit ribosomal RNA (ssrRNA), and the second on its mitochondrial DNA. For all samples, 5l of template DNA were used to initiate the primary reactions, and 1l from the resulting product was used to initiate the secondary reactions. For the ssrRNA-based detection, genus-specific oligonucleotide primers were used, rPLU1+rPLU2 in the primary amplification and rPLU3+rPLU4 in the secondary amplification, and for the positive samples a second round of secondary reactions was conducted to identify the species present, as described in a previously published protocol [61]. For the mitochondrial genome-based detection the primary reaction was carried out using MI-OF4A 5-GATGGAAACAGCCGGAAAG and MI-NR4 5- ATACAGTCCCAGCGACAGC, and the secondary reaction with GS-F4A 5- ATTAAAGGAACTCGACTGGCC and MI-NR4. The enzyme used was Phusion High Fidelity Taq polymerase (New England Biolabs, USA) and the reaction conducted in the buffer provided at final concentrations of 3mM for Mg2+ and 125 nM for each oligonucleotide primer. Following an initial denaturation of 5min at 95C, 30 cycles at 50C 15sec, 72C 15sec and 98C 10sec were carried out for the first reaction and 35 cycles at 65C 15sec, 72C 15sec and 98C 10sec for the second reaction. In the final cycle the extension was carried out for 5min before bringing the reaction to room temperature. The products were visualized by ethidium bromide staining after electrophoresis on a 3% agarose gel.

### Statistical analysis

Wilcoxon tests were used for paired samples when results of the same day were compared according to CO_2_ use, trap height, feeding site versus nesting site. To compare medians of the samples, MannWhitney for independent samples were used since normality tests were usually not passed (two-tailed P value). Non-parametric correlations between variables were measured with Spearman r (two-tailed P value).

## Results

### Potential vectors of *Plasmodium* species of chimpanzees

In Kanyawara, a total of 245 female *Anopheles* were collected in 113 traps over 151 nights of trapping (72 during the dry season and 79 during the rainy season), and in Kanyanchu 64 female *Anopheles* were caught in 36 traps over 43 nights of trapping. The highest number of female *Anopheles* found in a single trap was 28 in Kanyawara and 10 in Kanyanchu, and that of specimens caught on any one day (22 March 2007) was 34 in Kanyawara and 13 in Kanyanchu (29 May 2009), and these belonged to four species. In Kanyawara, a total 10 species of *Anopheles* were caught, and nine could be identified, while only three species were collected in Kanyanchu. Four of the species belonged to the sub-genus *Anopheles* and five to the sub-genus *Cellia* (51% of *Anopheles* vs 49% *Cellia*). Five of the nine known series (*Myzorhynchus, Christya, Neomyzomya, Myzomya, Cellia*) are represented (Table2).

**Table 2:** ***Anopheles *species captured and identified between January 2006 and January 2008**

**Genus *Anopheles***			
**Sub-genus*Anopheles***		**Sub-genus*Cellia***	
***Christya***	*A. implexus*	***Myzomya***	*A. demeilloni*
***Myzorhynchus***	*A. paludis*		*A. harperi*
	*A. ziemanni*		*A. marshallii*
	*A. obscurus*	***Neomyzomya***	*A. vinckei*
***Anopheles***		***Cellia***	*A. squamosus*
		***Neocellia***	
		***Pyretophorus***	
		***Paramyzomya***	

**Figure 1:**
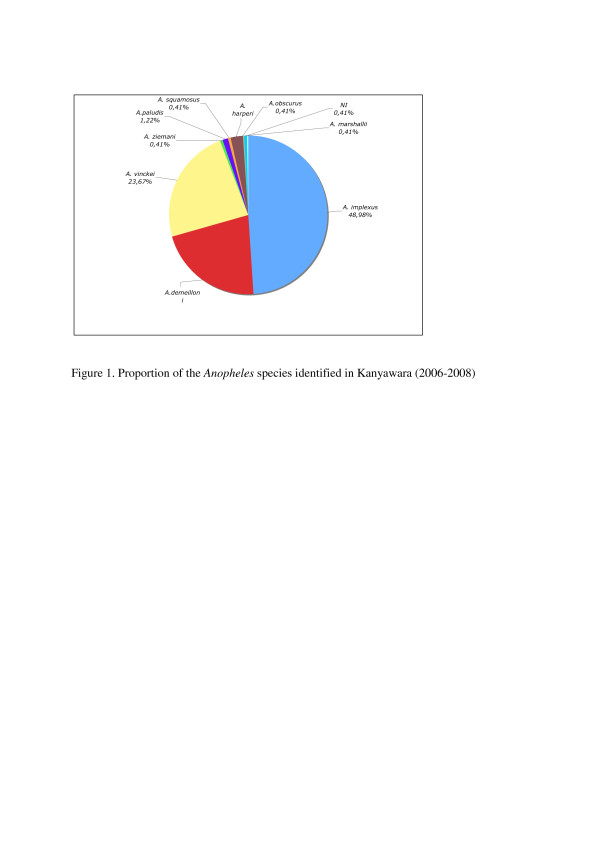
**Proportion of the *Anopheles *species identified in Kanyawara (2006-2008)**.

The three species most frequently collected were *A. implexus* (Kanyawara: 120/245, 49%; Kanyanchu: 42/64, 66%), *A. vinckei* (Kanyawara: 58/245, 24%; Kanyanchu: 15/64, 23%), and *A. demeilloni* (Kanyawara: 53/245, 22%; Kanyanchu: 6/64, 9%) (Figure1). In Kanyawara, *A implexus* were collected throughout the two years (21/24months), while *A. demeilloni* and *A. vinckei* were only collected during 13 and 12months of the study, respectively. The month when collections were most abundant (47 individuals) was August 2007, and up to five species were collected in July, October and December 2006.

### Factors that influenced the presence of *Anopheles* in the home range of chimpanzees

*Anopheles* females were caught in 72 out of the 300 traps placed in Kanyawara and in 30 of the 83 traps in Kanyanchu. These anophelines were further analysed with respect to the following factors:

### Meteorological factors

The temperature was significantly higher and the hygrometry lower in Kanyanchu (n=36 nights) compared to the dry season of collection in Kanyawara (n=70 nights) (morning temperature: Kanyawara: 16C Kanyanchu : 18.3C MannWhitney Test P<0.005; evening temperature : Kanyawara: 20C Kanyanchu : 22.1C MannWhitney Test P<0.001; morning hygrometry : Kanyawara: 89% Kanyanchu : 81% MannWhitney Test P=0.078; evening hygrometry : Kanyawara: 82.7% Kanyanchu : 75.5% MannWhitney Test P=0.011). Humidity was found to affect trapping success. In Kanyawara, hygrometry was significantly higher for traps in which females *Anopheles* were caught than for those found empty in the evening (Kanyawara: 78% empty traps *vs* 83% traps with *Anopheles*, MannWhitney test, P=0.0009;), or in the morning (Kanyawara: 85% empty traps *vs* 90% traps with *Anopheles*, MannWhitney test P<0.0001;). In Kanyanchu, no significant relation was found but the hygrometry was lower compared to Kanyawara (Kanyanchu: evening 76.9% empty traps *vs* 73.1% traps with *Anopheles* MannWhitney Test P=0.28; morning: 80.7% empty traps *vs* 81.3% traps with *Anopheles*, MannWhitney Test P=0.97). For both empty traps and those with *Anopheles*, the temperatures recorded did not differ significantly (Kanyawara: evening catches 20.4C for empty traps *vs* 20.2C for traps with *Anopheles*, MannWhitney test P=0.35; Kanyanchu: empty 21.9C with *Anopheles* 22.1C, MannWhitney Test P=0.287; Kanyawara: morning catches 16.1C for empty traps *vs* 16.1C for traps with *Anopheles*, MannWhitney test P=0.38, Kanyanchu empty 18.8C; with *Anopheles* 18.0C, MannWhitney Test P=0.96).

### Ecological factors

Mosquitoes, and female *Anopheles* in particular, were significantly more abundant in traps placed in the forest compared to those located in the villages, both in Kanyawara and Kanyanchu (mosquito scores for Kanyawara: forest=1.56, village=0.85, MannWhitney Test P<0.0001; number of female *Anopheles* per trap: forest=0.66, village=0.10, MannWhitney Test P=0.0092; for Kanyanchu mosquito score: forest=1.11; village=0.4 MannWhitney Test P=0.0024; number of female *Anopheles*/trap: forest =1.23; village =0.06, MannWhitney Test P=0.000097). *Anopheles implexus* and *Anopheles marshalli* were the only species collected in the villages.

### Trap type

**Figure 2:**
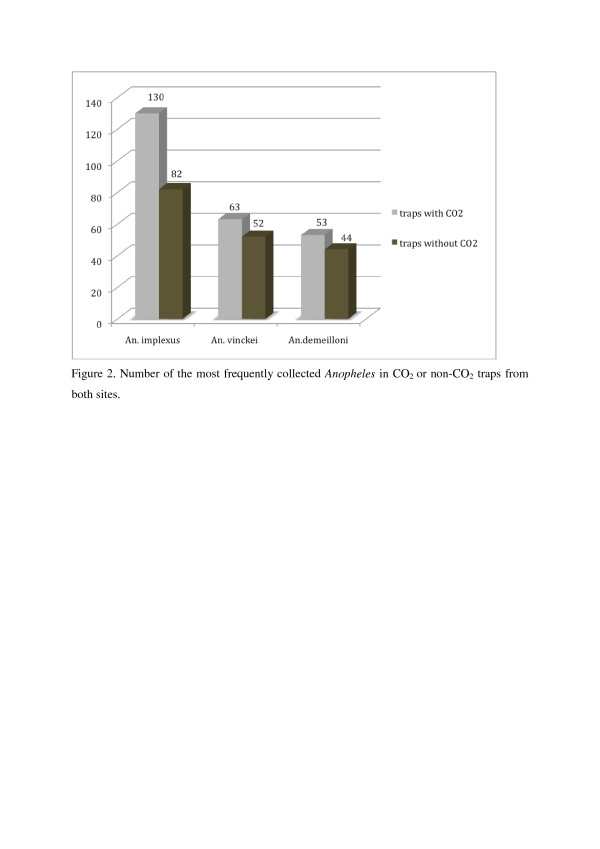
**Number of the most frequently collected *Anopheles *in CO**_**2 **_**or non-CO**_**2 **_**traps from both sites.**

Trapping-methods did not significantly affect trapping success. CO_2_ as compared to non-CO_2_ traps tended to attract more mosquitoes in general and female *Anopheles* in particular, but the difference was not significant in either communities. *Anopheles implexus* were slightly more attracted by the presence of CO_2_ than *A. vinckei* and *A. demeilloni* (Figure2).

### Variation in vector distribution between chimpanzee feeding and nesting sites

The mean altitude of the chimpanzee feeding sites, nesting sites and villages, where traps were set, was significantly higher for Kanyawara compared to that of Kanyanchu (unpaired *t* test : Kanyawara : 1,506m, n=106; Kanyanchu : 1,260m, n=38P<0.0001). The mean altitude of the chimpanzee feeding sites was lower than that of their nesting sites (feeding site=1506m *vs* nesting site =1516m, Wilcoxon test for 28 pairs of days 1, eight pairs excluded because equal, P=0.04, r Spearman=0.53, P=0.0004). The mean altitude of traps containing female *Anopheles* was lower (Kanyawara: 1,502m, n=65; Kanyanchu 1,257m, n=28) than that of empty traps (Kanyawara: 1,512m, n=246; Kanyanchu 1,269m, n=53), however, the difference between the medians was not significant (respectively MannWhitney Test P=0.1 and 0.22).

### Positional factors

The nest height was not significantly different between two sites during the study periods (Kanyawara 9.1m, n=74; Kanyanchu 8.1m, n=30; unpaired *t* test P=0.12). In Kanyawara, the number of chimpanzee nests in a nesting site was negatively correlated with mosquito abundance (r=0.18; P=0.01).

**Figure 3:**
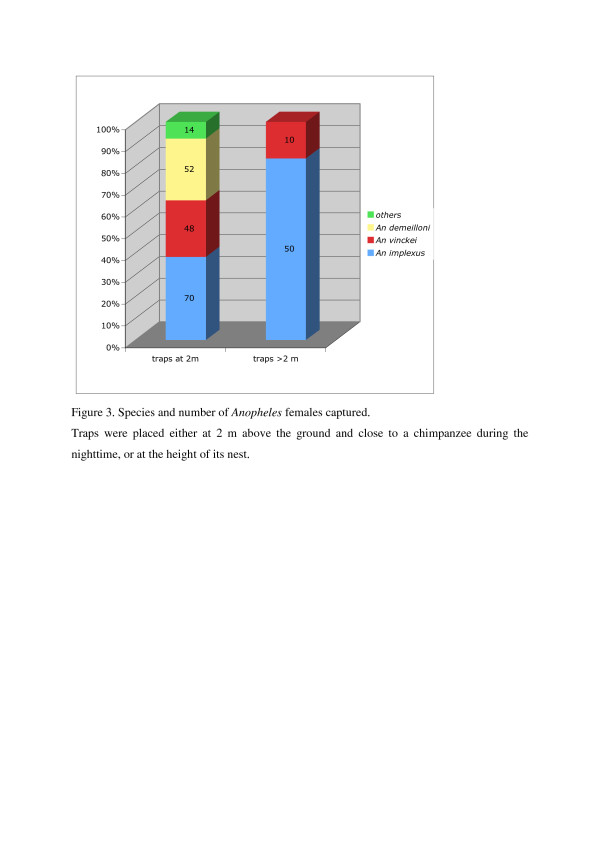
**Species and number of *Anopheles *females captured.** Traps were placed either at 2m above the ground and close to a chimpanzee during the night, or at the height of its nest.

Considering the two sites together, mosquito scores and trap height were negatively correlated (r=0.24, P<0.0001, n=416). Species composition varied with trap height and thus, nest height. Only two species were present, with *A. implexus* dominating (83% in Kanyawara; 58% in Kanyanchu) in traps placed close to the chimpanzees and at their nest heights (between 3 to 17m above ground). By contrast, in traps placed at 2m, 10 species were present and *A. implexus* represented only 38% of the specimens in Kanyawara, though 77% in Kanyanchu (Figure3).

### Molecular detection of *Plasmodium* parasites in humans and mosquitoes

*Plasmodium* parasites could not be detected in either duplicate amplification reactions of any of the DNA template purified from the female *Anopheles* samples collected, either when using the primers targeting the ssrRNA or those targeting the mitochondrial DNA. On the other hand, many of the blood samples (60%) collected from the humans working in or within 1km of the forest where the chimpanzee communities were found to harbour malaria parasites. Of the 74 human blood samples collected, 44 were found positive for *Plasmodium* parasites. *P. falciparum* was found in 80% of the positive samples and as a mixed infections in another 16%. The parasite species present were *P. falciparum* only in 30 samples, *P. falciparum*+*P. ovale* in five, *P. falciparum*+*P. malariae* in one, and *P. ovale* only in a single sample. The species could not be determined in the seven remaining samples, most likely because the amount of DNA was limiting.

## Discussion

Over a period of 28months, 309 female *Anopheles*, from 10 species, were collected in 383 traps placed in the habitat of two chimpanzee communities (Kanyawara and Kanyanchu) in Western Uganda. The number of species caught was consistent with other studies conducted in tropical areas: 10 species were collected in Malaysia in eight nights [62]. The number of anopheline mosquitoes caught might be considered to be quite low as compared to the numbers obtained in some studies conducted in human and livestock environments. For example, in a 1.5-year study conducted with light traps in Kenya, about 15,000 and 60,000 female *Anopheles* from only four species were collected inside and outside houses, respectively [63]. On the other hand, when the mosquito surveys were conducted in an environment similar to that where the chimpanzees live, namely cool highlands, the number of *Anopheles* collected is often very low: for instance using the same methodology and sampling protocols, 107 *Anopheles* were collected at Kyenjojo (about 20km to Kibale NP) as compared to the 10,127 *Anopheles* collected in Tororo, a dry savannah grassland area [64].

Ultimately the ecology and host preference of the *Anopheles* species identified in the home range of chimpanzees remain poorly documented. However, the meager observations do not exclude a role in malaria transmission. Only one of these species, *Anopheles paludis*, has been incriminated as an important vector of human malaria [65], though this role appeared to vary with geographical location. The forest species *A. implexus* has been described as an anthropophilic taxon, and in 1960, Lips found one female infected with malaria out of 1,200 dissected. Breeding places were usually described to be swamps, stagnant shady drains, water containing dead leaves and elephant tracks [66], but Lambrecht [67] failed to find any larva or egg in the forest gallery where adults where collected despite repeated efforts. *Anopheles obscurus*, commonly found in forests, does not bite humans but was found carrying malaria oocysts [68,69]. *Anopheles squamosus*, abundant from the peak to the end of the rainy season [70], is close to *Anopheles pharaoensis* but its role in human malaria transmission does not seem to be major. *Anopheles ziemani* is mostly described as a zoophilic species but has nevertheless been implicated in human malaria transmission [71]. *Anopheles demeilloni* lives in altitude (700 to1,800m), as does *Anopheles harperi* a rather zoophilic and exophilic species. *Anopheles vinckei* is poorly described and its distribution is limited to Oriental Kivu in Democratic Republic of Congo and Western Uganda.

Three *Anopheles* species (*A. implexus, A. vinckei* and *A. demeilloni*) represented more than 94% of the catch but only one, *A. implexus*, was dominant in the vicinity of the chimpanzees nests where it was consistently captured (21months over 24 of study). In a previous study [12], blood samples were obtained serendipitously from three Kanyawara community chimpanzees: one was injured in the course of a fight in September 2006, one as it was released from a poachers snare in October 2006, and the third in the course of a post-mortem in January 2007. Molecular analysis of the genomic DNA prepared from these samples revealed that all these three chimpanzees sampled randomly, within the study-period of the present survey, were infected with multiple species of *Plasmodium*[12]. Given that the Kanyawara community comprises only 44 individual chimpanzees, the presence of infections in three strongly suggests a very high prevalence of malaria in these apes, and this can only be due to high transmission rates, chronic persistence of parasites over long durations, or both. Observations of mixed infections in wild-caught chimpanzees are common as are sub-patent infections that persist for many years [15,16]. The prevalence of infection was high in the human blood samples analyzed, with *Plasmodium* parasites detected using the same PCR assays in 44 of the 74 (60%) persons sampled.

*Plasmodium* parasites could not be detected by PCR amplification in any of the *Anopheles* mosquitoes caught. Given the very low numbers of mosquitoes that were actually caught in the vicinity of the chimpanzee nests, this was to be expected since the infection rates observed in mosquitoes are usually low (< 1%) as are the parasite burdens (an average of one oocyst) even in mosquitoes caught in areas of high malaria endemicity. Ideally, one can optimize the chances to uncover the mosquitoes that transmit the parasite by collecting specimens that have fed on the chimpanzees, but this is impossible to achieve for individuals in the wild. One can also minimize the possibility of degradation by processing the mosquito material for DNA extraction immediately after collection. In this manner, it will be possible to target studies of chimpanzee behaviour in relation to mosquito avoidance. However, such studies are technically and practically challenging to conduct.

The hypothesis tested in this study was that *Anopheles* abundance would be higher in areas of the chimpanzees home range that have lower altitude and are more humid. Furthermore, if diseases transmitted by anophelines were to impact on chimpanzee health, then the apes would be more likely to select nesting sites away from the wetter areas and at levels with the lowest *Anopheles* abundance. Although the data presented here could be interpreted to support this hypothesis, the numbers of mosquitoes caught were low. Several recent studies emphasized the strong and synergistic effects of microclimate and altitude on malaria risk in human population, especially in highland sites [35,53,72]. In such areas, the valleys and basin-like depressions were recognized as less desirable areas to live, people living in the valleys receive more infective bites under such ambient conditions and the human density in these foci was relatively lower [35]. In the present study, the number of *Anopheles* females caught varied with the altitude, temperature and hygrometry of the various sites where the traps were placed and the same patterns of choice for sleeping sites are observed in chimpanzees. Differences related to captures in the two close sites sampled (separated by less than 20km but characterized by different microclimates) are not surprising. Ernst *et al.*[53] conducted a study in a 16km^2^ area where elevation ranged from 1,829 to 2,132m and showed striking magnitude of the differences even within this small area (up to 39-fold differences in incidence between the sub-unit areas of highest and lowest incidence). In our survey, the study in Kanyawara, the site of highest altitude, wetter atmosphere and cooler temperature, monitored during both rainy and dry seasons underlined the effects of climatic and spatial factors on the trap yields.

The low abundance of adult mosquitoes caught in the chimpanzees night environment might be in part explained by aspects of chimpanzee behaviour that lead to a reduction of exposure to malaria vectors. Chimpanzees are mobile and every evening they build a night nest in a new location within a large home range, about 20km^2^ in the present study-sites. In addition, even if chimpanzees are social, their population density is low (2.4 individuals/km^2^) and their system of fission-fusion prevents a high concentration of chimpanzees in a same nesting site at a same time. In Kibale NP, wet areas are usually found in the valley where swamps are frequent and footprints of elephants are very abundant. In human communities within a small area of 16km^2^, proximity to forest and swamp have both been associated with significant increased vector density: vector density has been shown to cluster in low-lying swampy areas [53]. The present results indicated that chimpanzees build nests at a higher altitude than the sites where they feed, suggesting that they prefer nesting site above the wet valley. A topographic preference of chimpanzees for nesting on ridges and shoulders was also noted by Furuichi and Hashimoto [73]. The negative correlation observed between nest number and mosquito abundance is consistent with these findings, although it cannot be exclude that mosquitoes might be more attracted by chimpanzees than by the traps. Nonetheless, the higher the nest site, the less diverse were the species of *Anopheles* encountered. There are many factors, such as predation pressure, body size or comfort, that have been proposed to influence the construction and selection of sleeping shelters and their height by great apes [74,75]. It was even suggested that nest construction could explain the cognitive evolution of hominoids through long-term memory consolidation related to higher quality sleep [76]. The present survey suggests that mosquito avoidance should be also considered as a factor in the selection of the nest location and height. This is especially relevant in Kibale NP where no predators exist and where chimpanzees are not hunted. Provided that the negative correlation between nest number/height and mosquito abundance does not reflect a higher attraction of mosquitoes to chimpanzees than to the traps, it would appear that chimpanzees select a nesting site where relatively few biting mosquitoes occurred. Moreover, mosquitoes are vectors of other diseases, including arboviral infections, that affect great apes and thus that can have significant impact on mortality and morbidity of wild primate populations [77].

From a conservation point of view, it would be of great importance to collect mosquitoes in the different sites where chimpanzees live. This is of particular importance with respect to degraded forest and at the edge of the park sites, where the vector species and their infections might differ from those collected in the middle of the park. Data records extending back to 1903 indicate that the Kibale region has become moister [78], which would likely lead to an increase in mosquito abundance. It is possible that chimpanzees, which had been adapted over thousands of years to forest vectors and parasites, might face novel threats to their health as changing climate and land conversion force then to live in fragmented forests and to use more frequently the forest edge.

## Conclusions

Ten anopheline species were collected in the home range of chimpanzees living in Ugandan highlands. This number is comparable to that recorded in the course of other studies conducted in tropical areas of similar ecological characteristics. The endemicity of *Plasmodium* infection is likely to be as high in chimpanzees as it is in humans living in this area. Nonetheless, none of the 100 female *Anopheles* analysed were positive for *Plasmodium*. This is not unexpected because the total number of female *Anopheles* that was collected in the home range of the chimpanzees was low, and the infection rates normally observed in wild-caught *Anopheles* are usually low (less than 1%). The dominant species in the vicinity of chimpanzee nests was *A. implexus*, which makes it the most likely species to serve as a vector for the *Plasmodium* species of chimpanzees. Further sampling will be required to confirm or refute this. Chimpanzee nesting sites were located in higher and drier locations where the female *Anopheles* caught were less abundant than in the sites where the chimpanzees were feeding. Furthermore, the number of chimpanzee nests at a nesting site was negatively correlated with the mosquito abundance. The site that the chimpanzees choose for nesting every night might be selected in part so as to minimize contact with anopheline mosquitoes, and this in turn might lead to a reduced risk in acquiring a malaria infection.

## Competing interests

The authors declare that they have no competing interests.

## Authors contributions

SK conceived of the study, participated in the data collection, performed the *Anopheles* identification and the statistical analysis and drafted the manuscript. J-MK and FL participated in the data collection. MC participated in the data collection and in editing the manuscript. ST and MI carried out the molecular genetic studies. GS, ST and MI designed and carried out the molecular assays, GS also contributed to the editing of the manuscript. JMK participated in the design of the study. JCG participated in the design of the study, performed the *Anopheles* identification and contributed to the editing of manuscript. All authors read and approved the final manuscript.
